# The Law Society and Solicitors' Touts

**Published:** 1917-08-04

**Authors:** Reginald R. Garratt

**Affiliations:** Secretary. Royal Free Hospital, London, W.C.


### The Law Society and Solicitors' Touts.

*To the Editor of* The Hospital.

Sir,—In your editorial on page 332 of this week's Hospital I notice an insinuation that the Law Society might do something in the matter of touting solicitors. I have to inform you that the Law Society are fully aware of this practice, and for a long time past have done their best to stamp out this nuisance. If you communicate with the Law Society's solicitors, Messrs. Humphries and Son, you will doubtless find corroboration of this statement.—Believe me, yours truly,

Reginald R. Garratt, Secretary.

Royal Free Hospital, London, W.C. 1, July 27, 1917.

